# Machine learning–based gait analysis to predict clinical frailty scale in elderly patients with heart failure

**DOI:** 10.1093/ehjdh/ztad082

**Published:** 2023-12-20

**Authors:** Yoshifumi Mizuguchi, Motoki Nakao, Toshiyuki Nagai, Yuki Takahashi, Takahiro Abe, Shigeo Kakinoki, Shogo Imagawa, Kenichi Matsutani, Takahiko Saito, Masashige Takahashi, Yoshiya Kato, Hirokazu Komoriyama, Hikaru Hagiwara, Kenji Hirata, Takahiro Ogawa, Takuto Shimizu, Manabu Otsu, Kunihiro Chiyo, Toshihisa Anzai

**Affiliations:** Department of Cardiovascular Medicine, Faculty of Medicine and Graduate School of Medicine, Hokkaido University, Kita-15 Nishi-7, Kita-ku, Sapporo 0608638, Japan; Department of Cardiovascular Medicine, Faculty of Medicine and Graduate School of Medicine, Hokkaido University, Kita-15 Nishi-7, Kita-ku, Sapporo 0608638, Japan; Department of Cardiovascular Medicine, Faculty of Medicine and Graduate School of Medicine, Hokkaido University, Kita-15 Nishi-7, Kita-ku, Sapporo 0608638, Japan; Department of Cardiovascular Medicine, Faculty of Medicine and Graduate School of Medicine, Hokkaido University, Kita-15 Nishi-7, Kita-ku, Sapporo 0608638, Japan; Department of Cardiovascular Medicine, Faculty of Medicine and Graduate School of Medicine, Hokkaido University, Kita-15 Nishi-7, Kita-ku, Sapporo 0608638, Japan; Department of Cardiology, Otaru Kyokai Hospital, Hokkaido, Japan; Department of Cardiology, National Hospital Organization Hakodate National Hospital, Hokkaido, Japan; Department of Cardiology, Sunagawa City Medical Center, Hokkaido, Japan; Department of Cardiology, Japan Red Cross Kitami Hospital, Hokkaido, Japan; Department of Cardiology, Japan Community Healthcare Organization Hokkaido Hospital, Sapporo, Japan; Department of Cardiology, Kushiro City General Hospital, Hokkaido, Japan; Department of Cardiology, Kushiro City General Hospital, Hokkaido, Japan; Department of Cardiology, Kushiro City General Hospital, Hokkaido, Japan; Department of Diagnostic Imaging, Faculty of Medicine and Graduate School of Medicine, Hokkaido University, Sapporo, Japan; Faculty of Information Science and Technology, Hokkaido University, Sapporo, Japan; Technical Planning Office, INFOCOM CORPORATION, Tokyo, Japan; Technical Planning Office, INFOCOM CORPORATION, Tokyo, Japan; Technical Planning Office, INFOCOM CORPORATION, Tokyo, Japan; Department of Cardiovascular Medicine, Faculty of Medicine and Graduate School of Medicine, Hokkaido University, Kita-15 Nishi-7, Kita-ku, Sapporo 0608638, Japan

**Keywords:** Artificial intelligence, Machine learning, Gait analysis, Frailty, Heart failure

## Abstract

**Aims:**

Although frailty assessment is recommended for guiding treatment strategies and outcome prediction in elderly patients with heart failure (HF), most frailty scales are subjective, and the scores vary among raters. We sought to develop a machine learning–based automatic rating method/system/model of the clinical frailty scale (CFS) for patients with HF.

**Methods and results:**

We prospectively examined 417 elderly (≥75 years) with symptomatic chronic HF patients from 7 centres between January 2019 and October 2023. The patients were divided into derivation (*n* = 194) and validation (*n* = 223) cohorts. We obtained body-tracking motion data using a deep learning–based pose estimation library, on a smartphone camera. Predicted CFS was calculated from 128 key features, including gait parameters, using the light gradient boosting machine (LightGBM) model. To evaluate the performance of this model, we calculated Cohen’s weighted kappa (CWK) and intraclass correlation coefficient (ICC) between the predicted and actual CFSs. In the derivation and validation datasets, the LightGBM models showed excellent agreements between the actual and predicted CFSs [CWK 0.866, 95% confidence interval (CI) 0.807–0.911; ICC 0.866, 95% CI 0.827–0.898; CWK 0.812, 95% CI 0.752–0.868; ICC 0.813, 95% CI 0.761–0.854, respectively]. During a median follow-up period of 391 (inter-quartile range 273–617) days, the higher predicted CFS was independently associated with a higher risk of all-cause death (hazard ratio 1.60, 95% CI 1.02–2.50) after adjusting for significant prognostic covariates.

**Conclusion:**

Machine learning–based algorithms of automatically CFS rating are feasible, and the predicted CFS is associated with the risk of all-cause death in elderly patients with HF.

## Graphical Abstract

**Graphical abstract:**
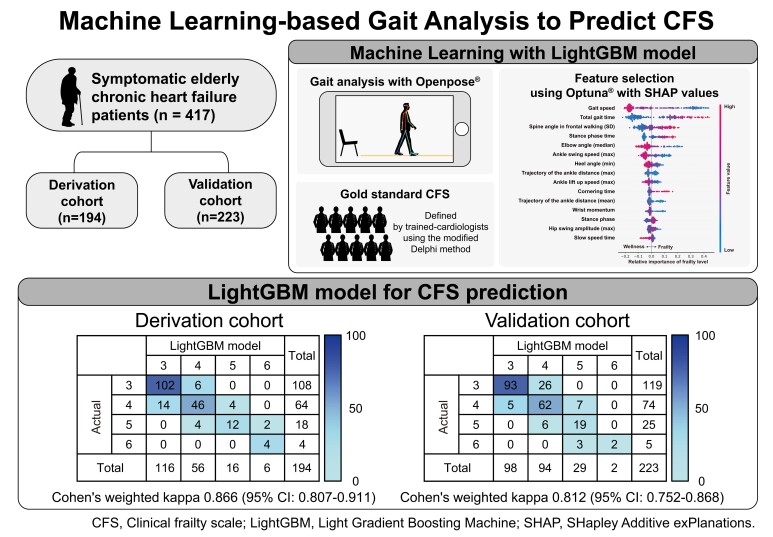

## Introduction

Heart failure (HF) is increasingly common worldwide despite the advances in medical therapy and devices, leading to a social problem of a HF pandemic.^1^ It is also a leading cause of hospitalization associated with poor clinical outcomes and high medical costs.^2,3^

Frailty is a geriatric syndrome with vulnerability to stressors caused by the accumulation of various physiological deficits.^4,5^ Many studies have shown a relationship between frailty and HF. For instance, 15–51% of patients with HF meet the diagnostic criteria for frailty, and the presence of HF also promotes frailty.^6,7^ Notably, frailty is a poor prognostic factor in patients with HF. In older adults (≥70 years of age) with HF, frailty assessed by the Cardiovascular Health Study definition for frailty phenotype (Fried’s criteria) was independently associated with 1-year mortality [hazard ratio (HR) 2.13, 95% confidence interval (CI) 1.07–4.23].^8,9^ Other studies also showed that frailty assessed by the Short Physical Performance Battery was significantly associated with 15 months mortality.^10,11^

One of the most widely used frailty assessment tools is the clinical frailty scale (CFS).^12,13^ The advantage of the CFS is that it is easy to use, provides an intuitive assessment, and requires no additional cost or effort for assessment. In addition, the frailty index (FI) can also be used for the assessment of frailty.^14^ As with the CFS, the FI is also associated with clinical outcomes in patients with HF.^15^ Nevertheless, it is necessary to evaluate at least 70 items in the FI, leading to a complexity of the frailty assessment. Although it is worthwhile to assess frailty in patients with HF, there is no consensus regarding how frailty should be measured. Therefore, this lack of consensus may be a major reason for not assessing frailty in clinical practice. In fact, the concordance rate between several frailty assessment tools is not sufficient.^12,16^ Furthermore, there are probably several inter-rater value errors, even in commonly used tools such as the CFS. The concordance rate of CFS assessment among specialists was reported to be high; however, it was considerably low between specialists and residents.^17–19^ These discordances and errors of commonly used frailty assessment tools could be caused by their subjective characteristics; therefore, the development of emerging objective tools for the assessment of frailty is warranted.

In the last decade, deep learning and machine learning techniques have shown promising image and video analysis capabilities in the field of medicine.^20–22^ Concerning motion analysis, these techniques can quickly and cost-effectively quantify and provide objective parameters of human movement such as gait. Gait analysis, including gait speed, gait width, and step width, has been used to evaluate patients’ frailty.^9,14,23^ Previous studies have shown that these parameters can be assessed by walking video analysis.^24,25^ However, there might be many more features that have not been exhaustively explored to characterize the gait and thus to predict the patients’ general condition. Accordingly, we aimed to develop and explore the feasibility of a machine learning–based automatic rating system for the CFS by focusing on walking video analysis in patients with HF.

## Methods

### Study design

This was a multi-centre (seven centres), observational, prospective study that included patients aged ≥75 years who were diagnosed with symptomatic chronic HF based on the diagnostic criteria from the current HF clinical guidelines.^26,27^ Patients with septic shock, acute coronary syndrome, acute pericarditis, receiving a left ventricular assist device or heart transplantation, open-heart surgery scheduled within 1 month, and the inability to walk independently were excluded. The included patients were divided into derivation (those from centre A: Hokkaido University Hospital) and validation (those from centre B: Japan Community Healthcare Organization Hokkaido Hospital, centre C: Japanese Red Cross Kitami Hospital, centre D: Otaru Kyokai Hospital, centre E: Sunagawa City Medical Centre, centre F: Hakodate National Hospital, and centre G: Kushiro City General Hospital) cohorts. This study was approved by the Institutional Review Board of each centre and is registered under the Japanese UMIN Clinical Trials Registration (UMIN000043390). The study conformed to the principles outlined in the Declaration of Helsinki. All the patients provided written informed consent to participate in the study.

### Study population

**Figure 1:**
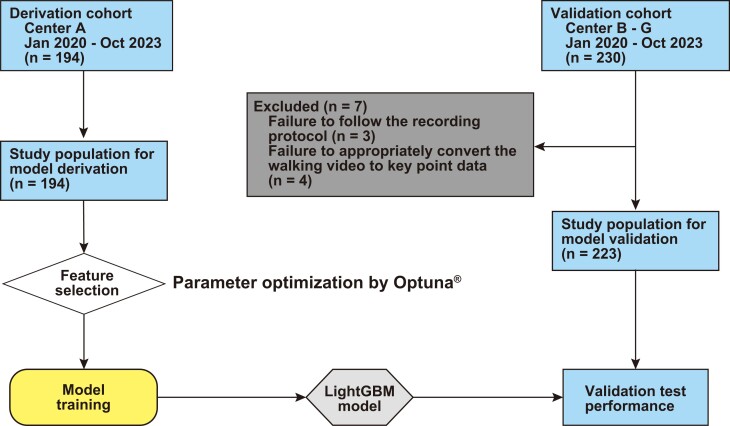
Flow diagram of the study. The validation test was performed using the light gradient boosting machine based on the extremely randomized trees regressor with the best-selected features. To explore the optimal combination of features, the derivation dataset was iteratively trained and explored using Optuna®. To calculate the internal test performance, the internal dataset was split into 90% training and 10% test, and the internal reliabilities were then obtained by a 10-fold cross-validation.

We examined 424 consecutive patients (derivation cohort, 194 patients; validation cohort, 230 patients) between January 2020 and October 2023. Patients whose walking parameters could not be appropriately obtained from the video in the validation cohort (*n* = 7) were excluded. Ultimately, 194 patients in the derivation cohort and 223 in the validation cohort were examined in this study (*Figure 1*).

### Clinical frailty scale rating

In general, the CFS consists of the following ratings: 1 (very fit), 2 (well), 3 (managing well), 4 (vulnerable), 5 (mildly frail), 6 (moderately frail), 7 (severely frail), 8 (very severely frail), and 9 (terminally ill).^13^ Nevertheless, CFS 1 and 2 are rated in patients with asymptomatic disease, and CFS 7 and over are rated in patients with an inability to walk without the assistance of caregivers.^13^ Thus, in the present study, all patients were evaluated as having CFS 3–6 because our study included only symptomatic patients with HF and excluded those who could not walk without the assistance of caregivers. We referred the CFS assessment, based on the obtained walking videos and clinical information, to 10 trained cardiologists certified by the Japanese Circulation Society (JCS). To minimize the inter-observer variability of CFS rating, the CFS evaluations by each rater were integrated using the modified Delphi method, which is a useful approach for the aggregation of varied opinions.^28^ We adopted their final assessment as the actual CFS. To compare the machine learning model with human raters, we selected three independent non-trained cardiologists who were post-graduate students without JCS certification.

### Development of gait analysis system

The gait analysis system begins with camera calibration, followed by enabling the extraction of key points from the human body and anomaly detection. Furthermore, after the extraction and selection of numerical parameters from the time series data of key points, the system enables the derivation of the final features for CFS prediction.

**Figure 2:**
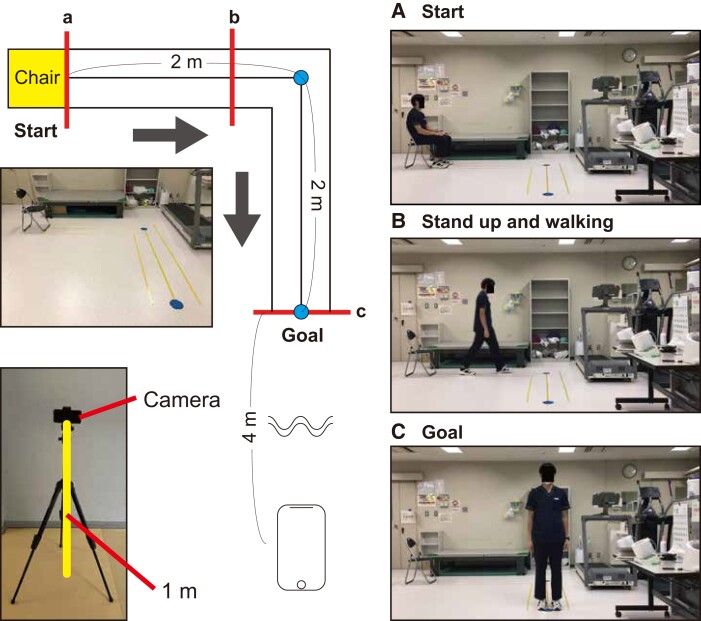
L-shaped walking course used in the study.

We developed an L-shaped walking course to obtain the patients’ gait parameters, as shown in *Figure 2*. Two blue markers were placed on the floor as distance reference points. The protocol for the walking video recording was as follows (see Supplementary material online, *Video S1*). After the patient sat on a chair, we started recording. The patient stood up from the chair and walked along the yellow line, turning to the right at the first marker (2 m away) and walked continuously, stopping at the second marker (further 2 m away), and we stopped the recording. Through this system, we could obtain ∼10–20 s of walking video, including a lateral view of the walk, turning motion, and frontal view of the walk. We recorded the walking video using a single smartphone camera (iPod touch®, 7th generation, Apple Inc., USA, memory capacity 128 GB, 30 frames per second at 1080p resolution), which was placed 4 m horizontally away from the second marker and 1 m vertically away from the floor.

The locations of the two markers were recognized using a single-shot multi-box detector (SSD), which is a deep learning–based object recognition technology.^29^ Specifically, the SSD was pre-trained on ImageNet, using the transfer learning technique, to recognize the marker appearances. The distance between the chair and first marker was 2 m. Camera calibration was performed according to the location of the marker.

All walking videos were converted to keypoint data by OpenPose®, a deep learning–based pose estimation library.^30^ OpenPose® is a deep learning–based keypoint detection algorithm for the human body. The keypoint data include the location and accuracy of the pose estimation for each frame of an input video. We used the BODY_25 model, which tracks the following 25 key points: the nose, neck, midhip, both sides of the shoulder, elbow, wrist, hip, knee, ankle, eye, ear, big toe, small toe, and heel. The original output data from OpenPose® are the estimation results for every frame; thus, the before/after frame relationship is not considered. Therefore, there are several misdetections in the left-foot and right-foot estimations. To solve this problem, an anomaly detection algorithm was used to detect the misestimations, and the data were corrected using spline interpolation from the previous and following values (see Supplementary material online, *Figure S1*). Specifically, the frame that includes the joint position change, which is >50 pixels in a positive direction or 35 pixels in a negative direction from the previous location, was defined as an abnormal frame. The location data of the bilateral foot (i.e. locations of the hip, knee, ankle, big toe, small toe, and heel) were deleted in the abnormal frame, and spline interpolation from the previous and following values was performed. From the locations of the 25 points and 2 floor markers, 123 features were calculated after camera calibration, and 5 features (age, sex, height, weight, and use of walking sticks) were added from the clinical information. Ultimately, 128 features were used for model training (see Supplementary material online, *Table S1*).

### Model development

In the derivation cohort, we used 128 features and developed a machine learning–based automatic rating system using the light gradient boosting machine (LightGBM) regressor model based on the extremely randomized trees model.^31,32^ The model development process is shown in *Figure 1*. First, we constructed a preliminary model using the derivation dataset to calculate the SHapley Additive exPlanations (SHAP) values.^33^ SHAP values >0 indicate a positive association and <0 indicate a negative association with frailty. The mean absolute SHAP value of each feature was used as the variable of importance. Second, the derivation datasets were split into 80% training and 20% test sets, and five-fold cross-validation was performed to estimate the temporary reliabilities between the actual and predicted CFSs. Third, feature selection was performed using Optuna®, an open-source hyper-parameter optimization framework (Preferred Networks, Inc., Tokyo, Japan). After the first hyper-parameter optimization, five-fold cross-validation and hyper-parameter optimization were repeated 50 times to explore the best feature selection. Finally, the LightGBM model was trained using the best-selected features in the derivation dataset and was tested in the validation dataset. To validate the internal reliabilities, the internal dataset was split into 90% training (as an internal dataset) and 10% test sets (as an external dataset), and the internal reliabilities were then obtained by 10-fold cross-validation. The LightGBM model output is a continuous value [frailty level (FL)], and the predicted CFS is the integer value of the FL. All estimation models were built using Python 3.6.

To protect patient privacy, we developed an offline system from video capture to CFS prediction, because the walking videos included patient privacy. The camera and small portable computer (Jetson AGX Xavier^TM^, NVIDIA®, USA) were connected with a wired cable, and the walking videos were uploaded from the camera to a portable computer without the internet. All processing (camera calibration, OpenPose®, anomaly detection algorithm, SSD, deletion of patient’s walking videos after converting the video to a key point data, and the LightGBM model for predicting the CFS) were performed on this computer, at each centre. Although the present study was a multi-centre study, patients’ personal information was not disclosed outside the study centres; thus, patient privacy was protected.

### Outcome measures

The primary outcome of interest was the agreement between the actual and predicted CFSs. The secondary outcome of interest was all-cause death stratified by the predicted CFS and FL.

### Statistical analysis

Continuous variables are presented as mean ± standard deviation when normally distributed and as median and inter-quartile range (IQR) when non-normally distributed. The differences between the two groups were compared using unpaired *t*-test or Mann–Whitney U-test for continuous variables and using χ^2^ test or Fisher’s exact test for dichotomous variables, when appropriate. The agreement between the actual and predicted CFSs was represented by Cohen’s weighted kappa and intraclass correlation coefficient (ICC), and 95% CIs were calculated using the bootstrap method. According to the magnitude of weighted kappa and ICC, the interpretation was as follows: ≤0.20, none; 0.21–0.39, minimal; 0.40–0.59, weak; 0.60–0.79, moderate; 0.80–0.90, strong; and >0.90, almost perfect.^34^ Cumulative incidence curves for all-cause death were generated to assess the prognostic significance of the predicted CFS. To evaluate the influence of the predicted CFS on all-cause death, we constructed four multivariable Cox proportional hazard models [Model 1: adjusted for age and sex; Model 2: adjusted for the Meta-Analysis Global Group in Chronic Heart Failure (MAGGIC) risk score; Model 3: adjusted for the MAGGIC risk score and N-terminal pro-brain natriuretic peptide (NT-pro BNP); and Model 4: adjusted for the MAGGIC risk score, NT-pro BNP, and serum albumin]. The MAGGIC risk score was calculated as follows.^35^ Total points were calculated based on age (every 10 years), male sex, body mass index (every 1 kg/m^2^ increase, up to 30 kg/m^2^), current smoking status, diabetes, systolic blood pressure (every 10 mmHg increase), New York Heart Association functional class, left ventricular ejection fraction (LVEF; every 5% increase, up to 40%), chronic obstructive pulmonary disease, HF duration >18 months, creatinine (10 up to 350 μmol/L), angiotensin-converting enzyme inhibitor/angiotensin II receptor blocker use, and beta-blocker use. A simple integer score was derived with a maximum score of 57. We tested the proportional-hazards assumption on the basis of Schoenfeld residuals after fitting Cox regression models. All tests were two-tailed, and a *P*-value <0.05 was considered statistically significant. All analyses were performed using STATA® MP64 version 16 (StataCorp, College Station, TX, USA).

## Results

### Baseline characteristics and gait parameters

Baseline characteristics of 194 patients from the derivation cohort and 223 patients from the validation cohort are summarized in *Table 1* and Supplementary material online, *Table S2*. Age was not significantly different (mean 82.3 vs. 82.5 years) between the derivation and validation cohorts. Patients from the derivation cohort were predominantly females [111 (57.2%) vs. 83 (37.2%)] compared with those from the validation cohort. Patients from the derivation cohort had a lower prevalence of ischaemic heart disease [23 (11.9%) vs. 61 (27.6%)], atrial fibrillation [94 (48.5%) vs. 130 (59.4%)], and smoking [68 (35.1%) vs. 118 (54.1%)]; lower haemoglobin, sodium, and creatinine levels; and higher NT-pro BNP level than those from the validation cohort. Regarding medications, patients from the derivation cohort were less frequently prescribed diuretics [117 (60.3%) vs. 166 (75.5%)], sodium-glucose transport protein-2 inhibitors [21 (10.8%) vs. 42 (19.1%)], and mineralocorticoid receptor antagonists [62 (32.0%) vs. 100 (45.5%)] compared with those from the validation cohort.

**Table 1:** Baseline patient characteristics of the derivation and validation cohorts

Variables	Derivation cohort	Validation cohort	*P*-value
	(*N* = 194)	(*N* = 223)	
Age, years	82.3 ± 4.9	82.5 ± 5.4	0.70
Female sex, *n* (%)	111 (57.2)	83 (37.2)	<0.001
BMI, kg/m^2^	22.8 (20.6–24.9)	22.1 (20.1–24.9)	0.36
SBP, mmHg	124 ± 20	124 ± 21	0.99
LVEF, %	60 (43–67)	55 (44–64)	0.095
NYHA III/IV, *n* (%)	37 (19.1)	40 (18.0)	0.80
Prior heart failure admission, *n* (%)	109 (56.2)	112 (50.7)	0.28
Smoking, *n* (%)	68 (35.1)	118 (54.1)	<0.001
Comorbidities, *n* (%)			
Ischaemic heart disease	23 (11.9)	61 (27.6)	<0.001
Prior stroke	27 (13.9)	33 (14.9)	0.78
Atrial fibrillation	94 (48.5)	130 (59.4)	0.030
Hypertension	136 (70.1)	140 (63.9)	0.21
Diabetes mellitus	59 (30.4)	76 (34.4)	0.40
COPD	13 (6.7)	13 (5.9)	0.84
Laboratory data			
Haemoglobin, g/dL	11.6 ± 1.7	12.3 ± 1.7	<0.001
Albumin, g/dL	3.8 ± 0.4	3.8 ± 0.4	0.30
Sodium, mEq/L	139 ± 2.8	140 ± 3.6	0.018
Potassium, mEq/L	4.1 ± 0.5	4.2 ± 0.6	0.19
Creatinine, mg/dL	0.96 (0.78–1.19)	1.08 (0.86–1.36)	<0.001
eGFR, mL/min/1.73 m^2^	49.0 ± 17	46.6 ± 17	0.15
NT-pro BNP, pg/mL	770 (377–1759)	1028 (528–2190)	0.008
Medications, *n* (%)			
Diuretics	117 (60.3)	166 (75.5)	0.001
ACE-inhibitors/ARBs	124 (63.9)	133 (60.7)	0.54
ARNI	8 (4.1)	18 (8.2)	0.11
Beta-blockers	122 (62.9)	158 (71.8)	0.058
SGLT-2 inhibitors	21 (10.8)	42 (19.1)	0.020
MRA	62 (32.0)	100 (45.5)	0.006
Statins	102 (52.6)	95 (43.2)	0.061

Continuous variables are presented as mean ± standard deviation if normally distributed and median (inter-quartile range) if not normally distributed. Categorical variables are presented as number of patients (%).

ACE, angiotensin-converting enzyme inhibitor; ARB, angiotensin II receptor blocker; ARNI, angiotensin receptor neprilysin inhibitor; BMI, body mass index; COPD, chronic obstructive pulmonary disease; eGFR, estimated glomerular filtration rate; LVEF, left ventricular ejection fraction; MRA, mineralocorticoid receptor antagonist; NT-pro BNP, N-terminal prohormone of brain natriuretic peptide; NYHA, New York Heart Association functional classification; SBP, systolic blood pressure; SGLT2, sodium-glucose cotransporter II.

**Figure 3:**
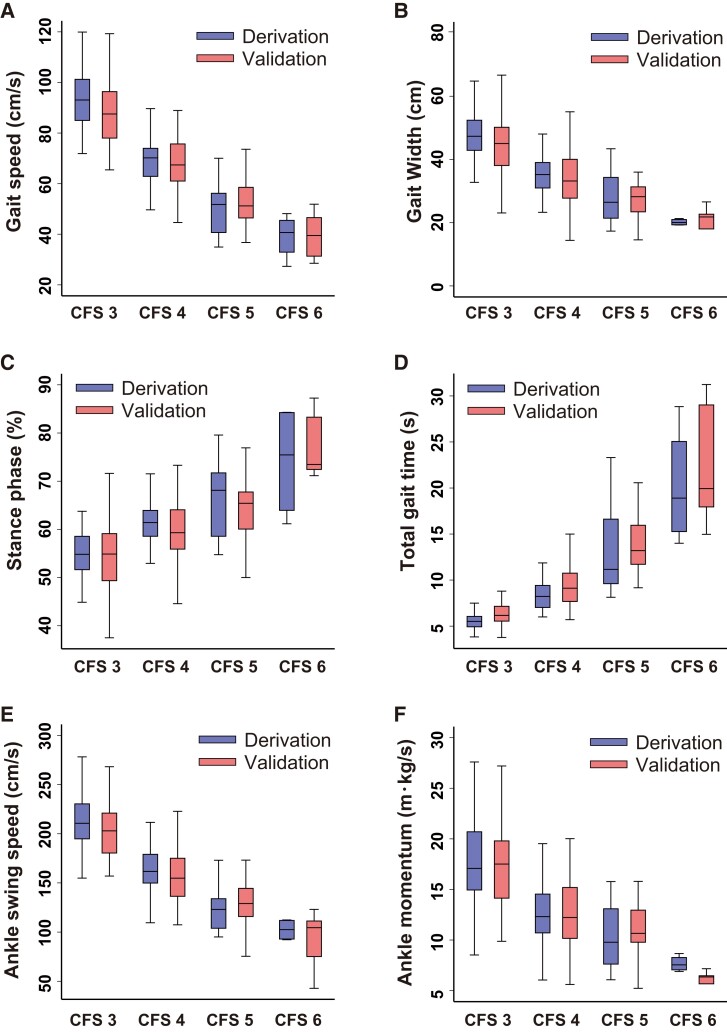
Comparison of the gait parameters between the derivation and validation cohorts across the clinical frailty scale categories. (*A*) Gait speed. (*B*) Gait width. (*C*) Stance phase. (*D*) Total gait time. (*E*) Ankle swing speed. (*F*) Ankle momentum.

The gait parameters obtained using the proposed application are listed in *Table 2*. Patients with higher CFS had higher total gait time, cornering time, stance phase, and prevalence of using walking sticks and lower gait speed, gait width, ankle swing speed and momentum, and wrist swing speed and momentum than those with lower CFS (*Figure 3A–F*).

**Table 2:** Gait parameters estimated using the machine learning model

	Overall	Category							
		CFS 3	LightGBM 3 (3 ≤ FL <4)	CFS 4	LightGBM 4 (4 ≤ FL <5)	CFS 5	LightGBM 5 (5 ≤ FL <6)	CFS 6	LightGBM 6 (6 ≤ FL <7)
Derivation cohort									
Number	194	108	116	64	56	18	16	4	6
Total gait time, s	6.57 (5.37–8.47)	5.52 (4.98–6.00)	5.60 (5.05–6.07)	8.23 (7.08–9.37)	8.37 (7.58–9.57)	11.2 (9.67–16.6)	10.9 (9.72–13.9)	18.9 (15.3–25.0)	19.2 (16.6–23.3)
Cornering time, s	2.98 (2.57–3.93)	2.63 (2.33–2.97)	2.67 (2.33–2.97)	3.73 (3.32–4.22)	3.83 (3.50–4.30)	5.13 (4.63–6.70)	4.65 (4.42–5.23)	7.93 (6.53–10.4)	7.93 (5.70–12.3)
Gait speed, cm/s	82.1 (66.8–94.5)	93.1 (85.2–101.0)	91.7 (84.8–100.6)	70.2 (63.1–73.8)	68.5 (62.1–73.0)	51.8 (40.9–56.0)	51.1 (43.0–55.3)	40.7 (33.1–45.3)	40.7 (34.9–48.2)
Gait width, cm	41.4 (33.2–48.3)	47.3 (43.0–52.3)	46.8 (42.8–51.7)	35.2 (31.1–38.9)	33.9 (31.0–38.1)	26.5 (21.6–34.2)	25.5 (22.2–29.6)	20.0 (19.5–20.8)	20.8 (19.7–27.5)
Stance phase, %	58.4 (54.0–62.3)	54.8 (51.7–58.5)	55.0 (52.0–58.5)	61.4 (58.7–63.8)	62.2 (59.1–63.9)	68.1 (58.7–71.6)	68.0 (59.0–70.4)	75.5 (64.0–84.1)	78.0 (66.9–84.0)
Ankle swing speed, cm/s	192 (159–216)	211 (195–230)	210 (196–229)	162 (150–179)	159 (149–170)	123 (104–133)	123 (105–131)	102 (93–111)	110 (95–112)
Wrist swing speed, cm/s	111 (86–143)	131 (104–158)	129 (104–157)	94 (75–116)	93 (75–114)	68 (58–78)	66 (56–80)	52 (46–56)	56 (48–63)
Ankle momentum, kg･m/s	15.0 (11.8–18.4)	17.1 (15.0–20.7)	17.4 (15.0–20.3)	12.3 (10.8–14.5)	12.0 (10.8–14.1)	9.8 (7.7–13.0)	8.5 (7.6–10.8)	7.5 (7.1–8.2)	8.2 (7.3–9.8)
Wrist momentum, kg･m/s	4.81 (3.36–6.17)	5.53 (4.39–7.33)	5.53 (4.39–7.31)	3.75 (3.08–5.09)	3.61 (3.12–5.09)	2.89 (2.28–3.78)	2.54 (2.18–3.51)	2.16 (1.81–2.31)	2.31 (2.09–3.01)
Walking stick, *n* (%)	20 (10.3)	0 (0.0)	0 (0.0)	5 (7.8)	6 (10.7)	11 (61.1)	9 (56.3)	4 (100)	5 (83.3)
Frailty level, points	3.74 (3.55–4.45)	3.57 (3.51–3.68)	3.57 (3.51–3.68)	4.35 (4.07–4.61)	4.45 (4.23–4.67)	5.32 (5.10–5.54)	5.32 (5.14–5.42)	6.07 (6.03–6.20)	6.07 (6.03–6.15)
Validation cohort									
Number	223	119	121	74	73	25	25	5	4
Total gait time, s	7.60 (6.03–10.50)	6.17 (5.60–7.10)	6.17 (5.60–6.97)	9.12 (7.73–10.7)	9.37 (8.13–11.1)	13.2 (11.8–15.9)	13.9 (12.4–15.9)	19.9 (18.0–29.0)	19.0 (16.8–24.5)
Cornering time, s	3.37 (2.77–4.43)	2.83 (2.43–3.47)	2.90 (2.43–3.37)	3.78 (3.30–4.77)	3.90 (3.37–4.77)	5.60 (4.83–6.40)	5.73 (4.83–6.57)	6.43 (4.40–13.07)	6.60 (5.27–9.92)
Gait speed, cm/s	77.6 (63.9–89.0)	87.5 (78.2–96.2)	87.6 (78.6–96.0)	67.4 (61.2–75.5)	67.3 (61.4–73.1)	51.2 (46.6–58.4)	49.1 (44.6–55.1)	39.5 (31.5–46.4)	45.7 (35.5–52.5)
Gait width, cm	38.1 (28.7–47.0)	45.0 (38.2–50.0)	45.7 (40.6–50.2)	33.2 (27.9–39.9)	33.2 (28.7–37.9)	28.2 (23.6–31.2)	25.7 (22.2–31.2)	21.8 (18.2–22.5)	22.2 (20.0–25.3)
Stance phase, %	58.1 (52.8–62.5)	54.9 (49.5–59.0)	55.0 (50.0–58.8)	59.3 (56.0–64.0)	59.7 (56.5–64.3)	65.4 (60.2–67.7)	65.6 (62.1–69.9)	73.5 (72.5–83.2)	71.8 (70.6–73.0)
Ankle swing speed, cm/s	178 (151–210)	203 (181–221)	204 (185–221)	155 (137–175)	155 (141–171)	129 (116–144)	123 (109–137)	104 (76–111)	108 (90–117)
Wrist swing speed, cm/s	110 (81–135)	128 (111–150)	129 (111–150)	92 (77–110)	93 (77–115)	67 (50–83)	66 (50–69)	50 (44–57)	47 (43–53)
Ankle momentum, kg･m/s	14.6 (11.2–18.2)	17.5 (14.2–19.8)	17.5 (14.8–20.0)	12.2 (10.2–15.1)	12.2 (10.3–14.6)	10.7 (9.8–12.9)	10.3 (8.6–11.1)	6.4 (5.7–6.4)	6.4 (6.0–8.2)
Wrist momentum, kg･m/s	4.76 (3.43–6.38)	5.70 (4.47–7.21)	5.75 (4.59–7.06)	3.93 (2.91–5.05)	3.92 (2.93–5.16)	3.10 (2.34–3.85)	2.70 (2.27–3.35)	1.53 (1.45–1.80)	1.69 (1.49–2.07)
Walking stick, *n* (%)	30 (13.5)	0 (0.0)	1 (0.8)	9 (12.2)	7 (9.6)	16 (64.0)	18 (72.0)	5 (100)	4 (100)
Frailty level, points	3.91 (3.60–4.55)	3.61 (3.54–3.82)	3.61 (3.54–3.78)	4.46 (4.06–4.69)	4.47 (4.20–4.69)	5.25 (5.08–5.50)	5.36 (5.20–5.53)	6.06 (5.98–6.26)	6.19 (6.10–6.30)

Continuous variables are presented as median (inter-quartile range). Categorical variables are presented as number of patients (%).

CFS, clinical frailty scale; FL, frailty level; LightGBM, light gradient boosting machine.

### Model performances

**Figure 4:**
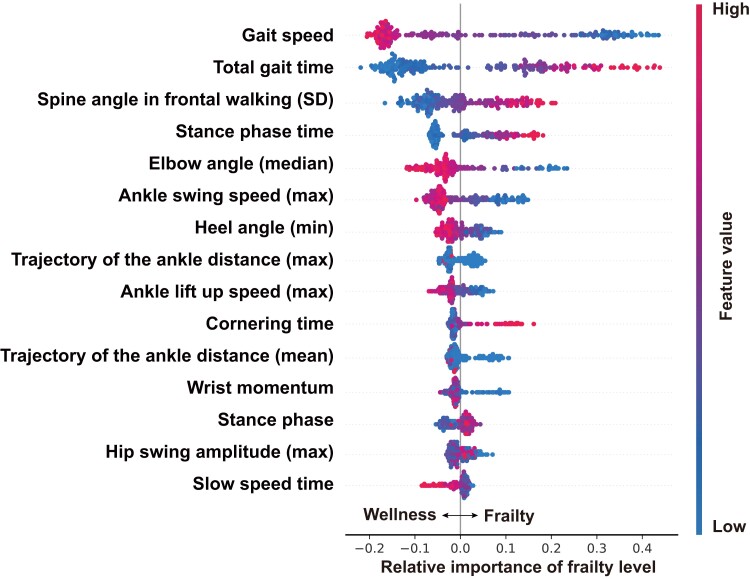
SHapley Additive exPlanations values of the 15 most important features of the light gradient boosting machine model.

**Figure 5:**
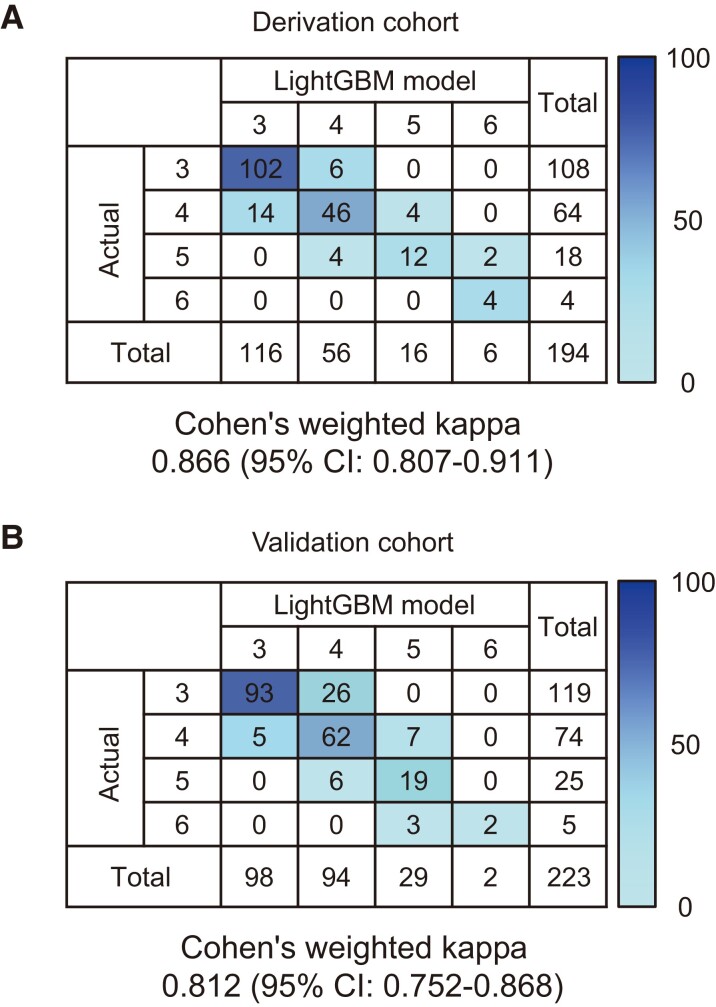
Correlation between the actual and predicted clinical frailty scales. (*A*) Derivation cohort. (*B*) Validation cohort. CI, confidence interval; LightGBM, light gradient boosting machine.

In the preliminary training, 45 parameters were selected according to the SHAP values to calculate the FL and estimate the CFS (see Supplementary material online, *Figure S2*). Fifteen most important features that the model used for prediction are depicted in *Figure 4*. In the derivation dataset, the LightGBM model showed an excellent agreement between the actual and predicted CFSs (weighted kappa 0.866, 95% CI 0.807–0.911; ICC 0.866, 95% CI 0.827–0.898; *Figure 5A* and *Table 3*). In the validation dataset, the LightGBM model also showed good agreement between the actual and predicted CFSs (weighted kappa 0.812, 95% CI 0.752–0.868; ICC 0.813, 95% CI 0.761–0.854; *Figure 5B* and *Table 3*). The LightGBM model achieved a higher agreement rate than the non-trained cardiologists (*Table 3*). None of the patients had a two-stage or over-estimation error in the derivation and validation datasets.

**Table 3:** Inter-rater reliability values of the light gradient boosting machine model and non-trained cardiologists with the actual clinical frailty scale

	Cohen’s weighted kappa		ICC	
	Kappa coefficient (95% CI^a^)	*P*-value	ICC (95% CI^a^)	*P*-value
Derivation cohort, *n* = 194				
LightGBM model	0.866 (0.807–0.911)	<0.001	0.866 (0.827–0.898)	<0.001
Non-trained cardiologist 1	0.867 (0.802–0.919)	<0.001	0.868 (0.818–0.904)	<0.001
Non-trained cardiologist 2	0.784 (0.703–0.848)	<0.001	0.784 (0.593–0.874)	<0.001
Non-trained cardiologist 3	0.740 (0.636–0.813)	<0.001	0.740 (0.462–0.875)	<0.001
Validation cohort, *n* = 223				
LightGBM model	0.812 (0.752–0.868)	<0.001	0.813 (0.761–0.854)	<0.001
Non-trained cardiologist 1	0.777 (0.693–0.842)	<0.001	0.774 (0.703–0.840)	<0.001
Non-trained cardiologist 2	0.677 (0.563–0.773)	<0.001	0.671 (0.562–0.756)	<0.001
Non-trained cardiologist 3	0.675 (0.595–0.745)	<0.001	0.669 (0.489–0.812)	<0.001

CFS, clinical frailty scale; CI, confidence interval; ICC, intraclass correlation coefficient; LightGBM, light gradient boosting machine.

^a^The 95% confidence intervals were calculated using the bootstrap method (1000 iterations).

### Survival analysis

During a median follow-up period of 391 (IQR 273–617) days, all-cause death occurred in 33 patients. Multivariable Cox regression analyses revealed that higher predicted CFS and FL were independently associated with all-cause death, even after adjusting for prognostic models (*Table 4*).

**Table 4:** Cox proportional hazard models for all-cause death

Model	Predicted CFS		Frailty level	
	HR (95% CI)	*P*-value	HR (95% CI)	*P*-value
Unadjusted	1.57 (1.98–2.29)	0.015	1.72 (1.15–2.59)	0.009
Model 1: age and sex	1.64 (1.10–2.45)	0.015	1.64 (1.10–2.45)	0.015
Model 2: MAGGIC risk score	1.60 (1.05–2.41)	0.026	1.79 (1.13–2.80)	0.013
Model 3: MAGGIC risk score and NT-pro BNP	1.65 (1.08–2.53)	0.021	1.75 (1.09–2.80)	0.020
Model 4: MAGGIC risk score, NT-pro BNP, and albumin	1.60 (1.02–2.50)	0.038	1.67 (1.03–2.72)	0.039

*P*-values for the proportional-hazard assumption on the basis of Schoenfeld residuals of the Models 1, 2, 3, and 4 are 0.63, 0.94, 0.41, and 0.56, respectively (predicted CFS) and for the Models 1, 2, 3, and 4 are 0.66, 0.91, 0.39, and 0.52, respectively (frailty level).

CFS, clinical frailty scale; CI, confidence interval; HR, hazard ratio; MAGGIC, Meta-Analysis Global Group in Chronic Heart Failure; NT-pro BNP, N-terminal pro-brain natriuretic peptide.

## Discussion

In this prospective study, we developed a machine learning–based automatic rating system to evaluate the CFS in elderly patients with HF. In addition, we evaluated the feasibility of the rating system by comparing it with the contemporary CFS rating system provided by trained cardiologists. We found that our automatic rating system using the LightGBM model has a strong reliability in comparison with trained cardiologists. Furthermore, the system proved to be as useful as a non-trained cardiologist for evaluating the CFS. We also found that higher predicted CFS and FL were independently associated with a risk of all-cause death, even after adjusting for prognostic covariates and a mortality prediction model.

In terms of the predicted CFS, our LightGBM model showed a strong agreement with the gold standard trained cardiologists (derivation cohort: weighted kappa 0.866 and ICC 0.866; validation cohort: weighted kappa 0.812 and ICC 0.813). Interestingly, these values were higher than those of the non-trained cardiologists (derivation cohort: weighted kappa 0.740–0.867 and ICC 0.740–0.868; validation cohort: weighted kappa 0.675–0.777 and ICC 0.669–0.774). Overall, our results indicate a good performance of the LightGBM model automatic rating system for estimating the CFS. One of the reasons for achieving good reliability of the predicted CFS was that we improved the training data (i.e. actual CFS) as much as possible using the modified Delphi method. The Delphi method is commonly used to obtain broad consensus among experts by determining the level of agreement on a given topic.^36^ To date, the definition of frailty is not clear and can differ in many aspects of developmental background, detailed components, and phenotype. Therefore, the Delphi method is suitable for developing high-quality training data. Notably, the validity of the content of a Delphi consensus will depend on the appropriate choice of the participating experts.^37^ Experts who participated in the study were well experienced in the field of cardiovascular medicine and were certified by the JCS. In addition, the level of participation was high: all the experts participated in each round, which supports the validity of the study as well as the final results.

For the development of the CFS automatic rating system, we found 15 key walking parameters, including gait speed, total gait time, ankle swing speed, and cornering time, based on the Shapley analysis without special cameras and/or body markers. Previous studies have shown that walking parameters can be analysed from the walking videos using machine learning models.^24,38,39^ However, in these studies, some special cameras and/or body markers were required; thus, the machine learning system is costly and available only in limited facilities. On the other hand, Vafadar *et al.*^25^ developed a marker-less system using a deep learning–based human pose tracking system that can estimate the spatiotemporal gait parameters within the range of the minimum detectable changes obtained using the marker-based reference system with special cameras. Furthermore, Park *et al.*^22^ developed a machine learning–based automated rating system for the two cardinal symptoms of Parkinson’s disease (i.e. resting tremor and bradykinesia) and confirmed the high reliability of their system compared with that of the manual gold standard ratings given by trained specialists.

In the present study, higher predicted CFS and FL were independently associated with a higher risk of all-cause death, even after adjusting for key prognostic confounders and a representative mortality prediction model. Several previous studies showed that frailty was a powerful predictor of morbidity and mortality, regardless of the frailty tool used (e.g. CFS, the Fried criteria, timed get-up-and-go test) and independent of age, comorbidities, HF symptoms, and severity.^40–43^ Sze *et al.*^43^ also found that the risk of hospital admissions and death increases as frailty status worsens. Given these information, the guidelines note that the treatment of frailty in HF should be multi-factorial and targeted to its main components and may include physical rehabilitation with exercise training and nutritional supplementation.^26^ A supervised, exercise-based, cardiac rehabilitation programme is also recommended in patients with HF and frailty.^26^ Most of the previous frailty indicators are based on classifications. Our automatic rating system can output not only the predicted CFS as a categorical value but also the FL as a continuous value. Evaluating frailty with continuous values may detect trajectories in frailty in a more sensitive manner than with classification indicators. Furthermore, the FL as detailed scores of the CFS (continuous value) would make it easier to compare before and after clinical intervention such as physical rehabilitation and nutritional supplementation.

There are several potential limitations of the present study that should be acknowledged. First, the walking video clips are two-dimensional images of the three-dimensional gait motion; thus, there may be several technical errors. Second, the actual CFS was determined by 10 trained cardiologists using the modified Delphi method. Because the Delphi method converges opinions based on majority voting, the results may be influenced by the tendencies and values of the evaluators. Third, the sample size used in our analyses, particularly the number of patients rated as CFS/LightGBM 5 or 6, was relatively small. Thus, the accuracy of the system may be limited. In addition, there were several differences in baseline patient characteristics between the derivation and validation cohorts. Fourth, our current methodology requires ∼5 min for estimating the FL and may be time consuming. Further studies developing an application that allows all processes, including walking video recording, prediction of the FL, and presenting the results output on a smartphone device, are required. Finally, owing to the relatively short-term follow-up (median 391 days), the association with long-term prognosis could not be investigated. Therefore, a prospective study with larger sample size is warranted to confirm the clinical implications of our CFS automatic rating system.

## Conclusions

In this study, we verified the feasibility of using a machine learning, vision-based automated rating system for estimating the CFS in patients with HF. We also confirmed the high reliability of this system, which was comparable with that of manual ratings by trained cardiologists. Furthermore, the automatically rated CFS was associated with the risk of all-cause death. The expansion into a more distributive system, confirmation of the prognostic implication of the automatically rated CFS, and its changes during clinical intervention such as physical rehabilitation would allow its use in clinical practice.

## Supplementary material

Supplementary material is available at *European Heart Journal – Digital Health*.

## Supplementary Material

ztad082_Supplementary_Data

## Data Availability

The data underlying this article cannot be shared publicly due to the privacy of individuals who participated in the study. The data will be shared on reasonable request with the corresponding author. No new data were generated or analysed in support of this research.
