# Supplementary material for: Machine learning–based gait analysis to predict clinical frailty scale in elderly patients with heart failure
Source: Eur Heart J Digit Health. 2023 Dec 20;5(2):152–62. doi: 10.1093/ehjdh/ztad082 (PMC10944685; doi:10.1093/ehjdh/ztad082)
Supplement: ztad082_Supplementary_Data [file ztad082_supplementary_data.zip › Supplementary Tables and Figures.docx]

**SUPPLEMENTARY MATERIALS**

**Machine Learning-based Gait Analysis to Predict Clinical Frailty Scale**

**in Elderly Patients with Heart Failure**

**Supplementary Online Content**

**Table of Contents:**

**Supplementary Table S1:** List of all features used in the study.

**Supplementary Table S2:** Baseline patient characteristics categorized by CFS.

**Supplementary Figure S1:** Anomaly detection algorithm and spline interpolation for misdetection.

**Supplementary Figure S2:** SHAP values of the selected features of the LightGBM model.

| **Supplementary Table S1. List of all features used in the study** | |
| --- | --- |
| Feature | Description |
| Age | Age, years |
| Ankle joint ROM | Range of motion of the ankle joint, degree |
| Ankle joint angle (max) | Maximum value of the ankle joint angle, degree |
| Ankle joint angle (min) | Minimum value of the ankle joint angle, degree |
| Arm swing amplitude (max) | Maximum value of the arm swing distance, cm |
| Slow speed time | Percent of duration of the ankle swing speed < the ankle swing speed (max)/10, % |
| Step width | Distance between both the ankles in frontal walking, cm |
| BV11 | Effect of the hip joint on the first factor of principal component analysis of the hip, knee, and ankle joints |
| BV12 | Effect of the knee joint on the first factor of principal component analysis of the hip, knee, and ankle joints |
| BV13 | Effect of the ankle joint on the first factor of principal component analysis of the hip, knee, and ankle joints |
| BV21 | Effect of the hip joint on the second factor of principal component analysis of the hip, knee, and ankle joints |
| BV22 | Effect of the knee joint on the second factor of principal component analysis of the hip, knee, and ankle joints |
| BV23 | Effect of the ankle joint on the second factor of principal component analysis of the hip, knee, and ankle joints |
| BV31 | Effect of the hip joint on the third factor of principal component analysis of the hip, knee, and ankle joints |
| BV32 | Effect of the knee joint on the third factor of principal component analysis of the hip, knee, and ankle joints |
| BV33 | Effect of the ankle joint on the third factor of principal component analysis of the hip, knee, and ankle joints |
| Cornering time | Duration of switch from horizontal to vertical walking, s |
| Bilateral ankle distance acceleration  (max) | Maximum acceleration value of the distance between both the ankles, cm/s^2^ |
| Bilateral ankle distance (mean) | Mean value of the distance between both the ankles, cm |
| Bilateral ankle distance (max) | Maximum value of the distance between both the ankles, cm |
| Bilateral ankle distance (variance) | Variance of the distance between both the ankles |
| Trajectory of the ankle distance  (max) | Maximum value of the trajectory of ankle distance, cm/s |
| Trajectory of the ankle distance  (mean) | Mean value of the trajectory of ankle distance, cm/s |
| Knee distance acceleration (max) | Maximum acceleration value of the distance between both the knees, cm/m^2^ |
| Knee distance (mean) | Mean value of the distance between both the knees, cm |
| Knee distance (max) | Maximum value of the distance between both the knees, cm |
| Knee distance (variance) | Variance of the distance between both the knees |
| Trajectory of the knee distance (max) | Maximum value of the trajectory of the knee distance, cm/s |
| Trajectory of the knee distance  (mean) | Mean value of the trajectory of the knee distance, cm/s |
| Eye angle (median) | Median value of the degree between horizontal and eyesight, degree |
| Height | Height, cm |
| Hip joint angle (mean) | Mean value of the hip joint angle, degree |
| Hip joint angle (valiance) | Variance of the hip joint angle |
| Knee joint ROM | Range of motion of the knee joint, degree |
| Knee joint angle (max) | Maximum value of the knee joint angle, degree |
| Knee joint angle (min) | Minimum value of the knee joint angle, degree |
| X coordinate of the midhip (mean) | Mean value of the X coordinate (horizontal component of the screen) of the midhip |
| X coordinate of the midhip (variance) | Variance of the X coordinate (horizontal component of the screen) of the midhip |
| Y coordinate of the midhip (mean) | Mean value of the Y coordinate (vertical component of the screen) of the midhip |
| Y coordinate of the midhip (variance) | Variance of the Y coordinate (vertical component of the screen) of the midhip |
| Walking stick | Use of a walking stick |
| Neck angle (mean) | Mean value of the neck angle, degree |
| Neck angle (variance) | Variance of the neck angle |
| Neck angle (max) | Maximum value of the neck angle, degree |
| Y coordinate of the neck (SD) | Standard deviation of the Y coordinate (vertical component of the screen) of the neck |
| Hip angle in frontal view (median) | Median value of the hip angle in frontal walking, degree |
| PC1 | The first factor of principal component analysis of the hip, knee, and ankle joints |
| PC2 | The second factor of principal component analysis of the hip, knee, and ankle joints |
| PC3 | The third factor of principal component analysis of the hip, knee, and ankle joints |
| Gait acceleration (max) | Maximum value of the gait acceleration, cm/s^2^ |
| Gait speed | Maximum value of the gait speed, cm/s |
| Pelvic bone slope (max) | Maximum value of the pelvic bone slope, degree |
| Pelvic bone slope (mean) | Mean value of the pelvic bone slope, degree |
| Pelvic bone slope (variance) | Variance of the pelvic bone slope |
| Ankle swing amplitude (max) | Maximum value of the right ankle swing amplitude, cm |
| Ankle swing amplitude (mean) | Mean value of the right ankle swing amplitude, cm |
| Ankle swing amplitude (min) | Minimum value of the right ankle swing amplitude, cm |
| Ankle swing amplitude (SD) | Standard deviation of the right ankle swing amplitude |
| Ankle lift down acceleration (max) | Maximum value of the right ankle lift down acceleration, cm/s^2^ |
| Ankle momentum | Right ankle momentum was calculated as following: Right ankle swing speed (max) * weight * 0.15, s･kg/m |
| Ankle swing acceleration (max) | Maximum value of the right ankle swing acceleration, cm/s^2^ |
| Ankle swing speed (max) | Maximum value of the right ankle swing speed, cm/s |
| Ankle lift up distance (max) | Maximum value of the right ankle lift up distance, cm |
| Ankle lift up acceleration (max) | Maximum value of the right ankle lift up acceleration, cm/s^2^ |
| Ankle lift up speed (max) | Maximum value of the right ankle lift up speed, cm/s |
| Gait width | Maximum value of the distance between the right and left ankles, cm |
| X coordinate of the right ankle  (mean) | Mean value of the X coordinate (horizontal component of the screen) of the right ankle |
| X coordinate of the right ankle  (variance) | Variance of the X coordinate (horizontal component of the screen) of the right ankle |
| Y coordinate of the right ankle  (mean) | Mean value of the Y coordinate (vertical component of the screen) of the right ankle |
| Y coordinate of the right ankle  (variance) | Variance of the Y coordinate (vertical component of the screen) of the right ankle |
| Big toe swing amplitude (max) | Maximum value of the right big toe swing amplitude, cm |
| Big toe swing amplitude (mean) | Mean value of the right big toe swing amplitude, cm |
| Big toe swing amplitude (min) | Minimum value of the right big toe swing amplitude, cm |
| Big toe swing amplitude (SD) | Standard deviation of the right big toe swing amplitude |
| Elbow swing amplitude (max) | Maximum value of the right elbow swing amplitude, cm |
| Elbow swing amplitude (mean) | Mean value of the right elbow swing amplitude, cm |
| Elbow swing amplitude (min) | Minimum value of the right elbow swing amplitude, cm |
| Elbow swing amplitude (SD) | Standard deviation of the right elbow swing amplitude |
| Elbow angle (median) | Median value of the right elbow angle, degree |
| Heel angle (min) | Minimum value of the right heel angle, degree |
| Hip swing amplitude (max) | Maximum value of the right hip swing amplitude, cm |
| Hip swing amplitude (mean) | Mean value of the right hip swing amplitude, cm |
| Hip swing amplitude (min) | Minimum value of the right hip swing amplitude, cm |
| Hip swing amplitude (SD) | Standard deviation of the right hip swing amplitude |
| Hip joint angle (max) | Maximum value of the right hip joint angle, degree |
| Hip joint angle (mean) | Mean value of the right hip joint angle, degree |
| Hip joint angle (variance) | Valiance of the right hip joint angle, degree |
| Knee swing amplitude (max) | Maximum value of the right knee swing amplitude, cm |
| Knee swing amplitude (mean) | Mean value of the right knee swing amplitude, cm |
| Knee swing amplitude (min) | Minimum value of the right knee swing amplitude, cm |
| Knee swing amplitude (SD) | Standard deviation of the right knee swing amplitude |
| Shoulder swing amplitude (max) | Maximum value of the right shoulder swing amplitude, cm |
| Shoulder swing amplitude (mean) | Mean value of the right shoulder swing amplitude, cm |
| Shoulder swing amplitude (min) | Minimum value of the right shoulder swing amplitude, cm |
| Shoulder swing amplitude (SD) | Standard deviation of the right shoulder swing amplitude |
| Wrist swing amplitude (max) | Maximum value of the right wrist swing amplitude, cm |
| Wrist swing amplitude (mean) | Mean value of the right wrist swing amplitude, cm |
| Wrist swing amplitude (min) | Minimum value of the right wrist swing amplitude, cm |
| Wrist swing amplitude (SD) | Standard deviation of the right wrist swing amplitude |
| Wrist momentum | Right wrist momentum was calculated as following: Right wrist swing speed (max) * weight * 0.08, s･kg/m |
| Wrist swing acceleration (max) | Maximum value of the right wrist swing acceleration, cm/s^2^ |
| Wrist swing speed (max) | Maximum value of the right wrist swing speed, cm/s |
| X coordinate of the right wrist  (mean) | Mean value of the X coordinate (horizontal component of the screen) of the right wrist |
| X coordinate of the right wrist  (variance) | Variance of the X coordinate (horizontal component of the screen) of the right wrist |
| Y coordinate of the right wrist  (mean) | Mean value of the Y coordinate (vertical component of the screen) of the right wrist |
| Y coordinate of the right wrist  (variance) | Variance of the Y coordinate (vertical component of the screen) of the right wrist |
| Sex | Sex |
| Shoulder slope (max) | Maximum value of the shoulder slope, degree |
| Shoulder slope (mean) | Mean value of the shoulder slope, degree |
| Shoulder slope (variance) | Variance of the shoulder slope |
| Spine angle in stand up (max) | Maximum value of the spine angle in stand up, degree |
| Spine angle in stand up (mean) | Mean value of the spine angle in stand up, degree |
| Spine angle in sitting | Value of the spine angle in sitting, degree |
| Spine angle in frontal walking  (mean) | Mean value of the spine angle in frontal walking, degree |
| Spine angle in frontal walking (SD) | Standard deviation of the spine angle in frontal walking |
| Spine angle in horizontal walking  (mean) | Mean value of the spine angle in horizontal walking, degree |
| Spine angle (max) | Maximum value of the spine angle, degree |
| Angle of spine and pelvis (max) | Maximum value of the angle between the spine and pelvis, degree |
| Angle of spine and pelvis (mean) | Mean value of the angle between the spine and pelvis, degree |
| Angle of spine and pelvis (variance) | Variance of the angle between the spine and pelvis |
| Stance phase | Percent of the stance phase, % |
| Stance phase time | Duration of the total stance phase, s |
| Swing phase | Percent of the swing phase, % |
| Swing phase time | Duration of the total swing phase, s |
| Total gait time | Duration between the first step and time when the patient stopped walking, time |
| Total motion time | Duration between the stand up and first step, time |
| Toe angle (min) | Minimum value of the toe angle, degree |
| Weight | Weight, kg |

ROM, range of motion; SD, standard deviation.

| **Supplementary Table S2.** **Baseline patient characteristics categorized by CFS** | | | | | | | | | | | | | | | | |
| --- | --- | --- | --- | --- | --- | --- | --- | --- | --- | --- | --- | --- | --- | --- | --- | --- |
| Variables |  | Clinical frailty scale | | | | | | | | | | | | | | |
|  |  | CFS 3 | | |  | CFS 4 | | |  | CFS 5 | | |  | CFS 6 | | |
|  |  | Derivation  cohort  (n = 108) | Validation  cohort  (n = 119) | *P* value |  | Derivation  cohort  (n = 64) | Validation  cohort  (n = 74) | *P* value |  | Derivation  cohort  (n = 18) | Validation  cohort  (n = 25) | *P* value |  | Derivation  cohort  (n = 4) | Validation  cohort  (n = 5) | *P* value |
| Age, years |  | 81.4 ± 4.5 | 81.2 ± 4.6 | 0.69 |  | 83.6 ± 4.7 | 83.0 ± 5.2 | 0.50 |  | 82.7 ± 6.1 | 86.3 ± 6.5 | 0.074 |  | 86.8 ± 5.0 | 89.8 ± 5.8 | 0.43 |
| Female sex, *n* (%) |  | 58 (53.7) | 39 (32.8) | 0.002 |  | 36 (56.3) | 30 (40.5) | 0.087 |  | 13 (72) | 12 (48) | 0.13 |  | 4 (100) | 2 (40) | 0.17 |
| BMI, kg/m^2^ |  | 23.1  (20.8-25.1) | 22.3  (20.3-25.1) | 0.36 |  | 21.9  (19.6-24.1) | 22.0  (19.8-24.6) | 0.80 |  | 23.6  (22.2-25.1) | 23.5  (21.3-24.8) | 0.43 |  | 23.1  (21.9-28.0) | 20.3  (18.5-21.8) | 0.14 |
| SBP, mmHg |  | 125 ± 20 | 125 ± 22 | 0.88 |  | 121 ± 19 | 125 ± 19 | 0.25 |  | 125 ± 23 | 117 ± 25 | 0.29 |  | 129 ± 13 | 123 ± 24 | 0.70 |
| LVEF, % |  | 60 (43-67) | 55 (43-65) | 0.21 |  | 54 (43-67) | 57 (46-64) | 0.97 |  | 63 (53-70) | 54 (43-64) | 0.11 |  | 71 (68-75) | 57 (57-61) | 0.032 |
| NYHA III/IV, *n* (%) |  | 15 (13.9) | 11 (9.2) | 0.30 |  | 16 (25.0) | 16 (21.9) | 0.69 |  | 5 (28) | 12 (48) | 0.22 |  | 1 (25) | 1 (20) | 1.00 |
| Prior heart failure admission, *n* (%) |  | 53 (49.1) | 62 (52.1) | 0.69 |  | 41 (64.1) | 34 (47.2) | 0.058 |  | 13 (72) | 12 (48) | 0.13 |  | 2 (50) | 4 (80) | 0.52 |
| Smoking, *n* (%) |  | 33 (30.6) | 72 (61.5) | <0.001 |  | 31 (48.4) | 35 (48.6) | 1.00 |  | 4 (22) | 10 (40) | 0.33 |  | 0 (0) | 1 (25) | 1.00 |
| Comorbidities, *n* (%) |  |  |  |  |  |  |  |  |  |  |  |  |  |  |  |  |
| Ischemic heart disease |  | 10 (9.3) | 37 (31.4) | <0.001 |  | 12 (18.8) | 21 (28.8) | 0.23 |  | 1 (6) | 3 (12) | 0.63 |  | 0 (0) | 0 (0) |  |
| Prior stroke |  | 13 (12.0) | 15 (12.6) | 1.00 |  | 8 (12.5) | 12 (16.7) | 0.63 |  | 5 (28) | 4 (16) | 0.46 |  | 1 (25) | 2 (40) | 1.00 |
| Atrial fibrillation |  | 50 (46.3) | 67 (57.3) | 0.11 |  | 35 (54.7) | 46 (63.0) | 0.38 |  | 9 (50) | 14 (56) | 0.76 |  | 0 (0) | 3 (75) | 0.14 |
| Hypertension |  | 76 (70.4) | 67 (56.8) | 0.039 |  | 44 (68.8) | 54 (75.0) | 0.45 |  | 12 (67) | 15 (63) | 1.00 |  | 4 (100) | 4 (80) | 1.00 |
| Diabetes mellitus |  | 28 (25.9) | 39 (33.1) | 0.25 |  | 24 (37.5) | 29 (39.7) | 0.86 |  | 6 (33) | 7 (28) | 0.75 |  | 1 (25) | 1 (20) | 1.00 |
| COPD |  | 6 (5.6) | 11 (9.2) | 0.32 |  | 5 (7.8) | 1 (1.4) | 0.099 |  | 2 (11) | 1 (4) | 0.56 |  | 0 (0) | 0 (0) |  |
| Laboratory data |  |  |  |  |  |  |  |  |  |  |  |  |  |  |  |  |
| Hemoglobin, g/dL |  | 11.8 ± 1.7 | 12.6 ± 1.7 | 0.001 |  | 11.4 ± 1.8 | 12.0 ± 1.7 | 0.035 |  | 11.0 ± 1.5 | 11.6 ± 1.2 | 0.16 |  | 10.2 ± 1.6 | 10.7 ± 1.5 | 0.64 |
| Albumin, g/dL |  | 3.8 ± 0.4 | 3.9 ± 0.4 | 0.18 |  | 3.7 ± 0.4 | 3.8 ± 0.5 | 0.55 |  | 3.7 ± 0.5 | 3.7 ± 0.4 | 0.97 |  | 3.8 ± 0.2 | 3.3 ± 0.3 | 0.033 |
| Sodium, mEq/L |  | 139.3 ± 2.5 | 140.0 ± 3.5 | 0.082 |  | 138.7 ± 3.1 | 139.8 ± 3.7 | 0.060 |  | 139.3 ± 3.1 | 140.3 ± 2.8 | 0.28 |  | 142.0 ± 1.4 | 136.5 ± 7.3 | 0.19 |
| Potassium, mEq/L |  | 4.1 ± 0.4 | 4.2 ± 0.5 | 0.39 |  | 4.1 ± 0.6 | 4.2 ± 0.7 | 0.27 |  | 4.0 ± 0.5 | 4.0 ± 0.4 | 0.88 |  | 4.2 ± 0.3 | 4.3 ± 0.2 | 0.36 |
| Creatinine, mg/dL |  | 0.97 (0.79-1.17) | 1.08 (0.85-1.31) | 0.017 |  | 0.96 (0.77-1.24) | 1.04 (0.87-1.36) | 0.12 |  | 0.94 (0.77-1.14) | 1.29 (1.04-1.47) | 0.020 |  | 0.96 (0.68-1.37) | 0.91 (0.77-1.04) | 1.00 |
| eGFR, mL/min/1.73 m^2^ |  | 49.4 ± 15.5 | 47.9 ± 15.8 | 0.48 |  | 48.2 ± 18.4 | 46.7 ± 19.5 | 0.64 |  | 50.2 ± 21.4 | 38.3 ± 12.6 | 0.027 |  | 44.9 ± 19.0 | 57.2 ± 12.9 | 0.32 |
| NT-pro BNP, pg/mL |  | 653 (328-1316) | 820 (419-1730) | 0.073 |  | 1090 (457-2024) | 1216 (594-3810) | 0.39 |  | 810 (236-2027) | 1408 (1036-2384) | 0.089 |  | 438 (209-796) | 990 (718-3206) | 0.083 |
| Medications, *n* (%) |  |  |  |  |  |  |  |  |  |  |  |  |  |  |  |  |
| Diuretics |  | 60 (55.6) | 82 (70.1) | 0.027 |  | 45 (70.3) | 57 (78.1) | 0.33 |  | 9 (50) | 23 (92) | 0.004 |  | 3 (75) | 4 (80) | 1.00 |
| ACE-inhibitors/ARBs |  | 66 (61.1) | 68 (58.6) | 0.79 |  | 42 (65.6) | 46 (63.0) | 0.86 |  | 13 (72) | 15 (60) | 0.52 |  | 3 (75) | 4 (80) | 1.00 |
| ARNI |  | 5 (4.6) | 9 (7.7) | 0.41 |  | 2 (3.1) | 5 (6.8) | 0.45 |  | 1 (6) | 4 (16) | 0.38 |  | 0 (0) | 0 (0) |  |
| Beta-blockers |  | 68 (63.0) | 85 (72.6) | 0.15 |  | 45 (70.3) | 53 (72.6) | 0.85 |  | 8 (44) | 18 (72) | 0.11 |  | 1 (25) | 2 (40) | 1.00 |
| SGLT-2 inhibitors |  | 12 (11.1) | 21 (17.9) | 0.19 |  | 8 (12.5) | 16 (21.9) | 0.18 |  | 0 (0) | 5 (20) | 0.064 |  | 1 (25) | 0 (0) | 0.44 |
| MRA |  | 34 (31.5) | 48 (41.0) | 0.17 |  | 24 (37.5) | 39 (53.4) | 0.086 |  | 4 (22) | 12 (48) | 0.12 |  | 0 (0) | 1 (20) | 1.00 |
| Statins |  | 53 (49.1) | 53 (45.3) | 0.59 |  | 38 (59.4) | 32 (43.8) | 0.087 |  | 9 (50) | 8 (32) | 0.34 |  | 2 (50) | 2 (40) | 1.00 |

Continuous variables are presented as mean ± standard deviation if normally distributed, and median (interquartile range) if not normally distributed. Categorical variables are presented as number of patients (%). BMI, body mass index; SBP, systolic blood pressure; LVEF, left ventricular ejection fraction; NYHA, New York Heart Association functional classification; COPD, chronic obstructive pulmonary disease; eGFR, estimated glomerular filtration rate; NT-pro BNP, N-terminal prohormone of brain natriuretic peptide; ACE, angiotensin-converting enzyme inhibitor; ARB, angiotensin II receptor blocker; ARNI, angiotensin receptor neprilysin inhibitor; SGLT2, sodium-glucose cotransporter II; MRA, mineralocorticoid receptor antagonist.

**Supplementary Figure S1: Anomaly detection algorithm and spline interpolation for misdetection**

An abnormal frame was defined as: the frame that included the joint position change, which was more than 50 pixels in a positive direction or 35 pixels in a negative direction from the previous location. The location data of the bilateral foot (i.e., locations of the midhip, knee, ankle, big toe, small toe, and heel) were deleted in the abnormal frame, and spline interpolation was performed using the previous and following values.

**
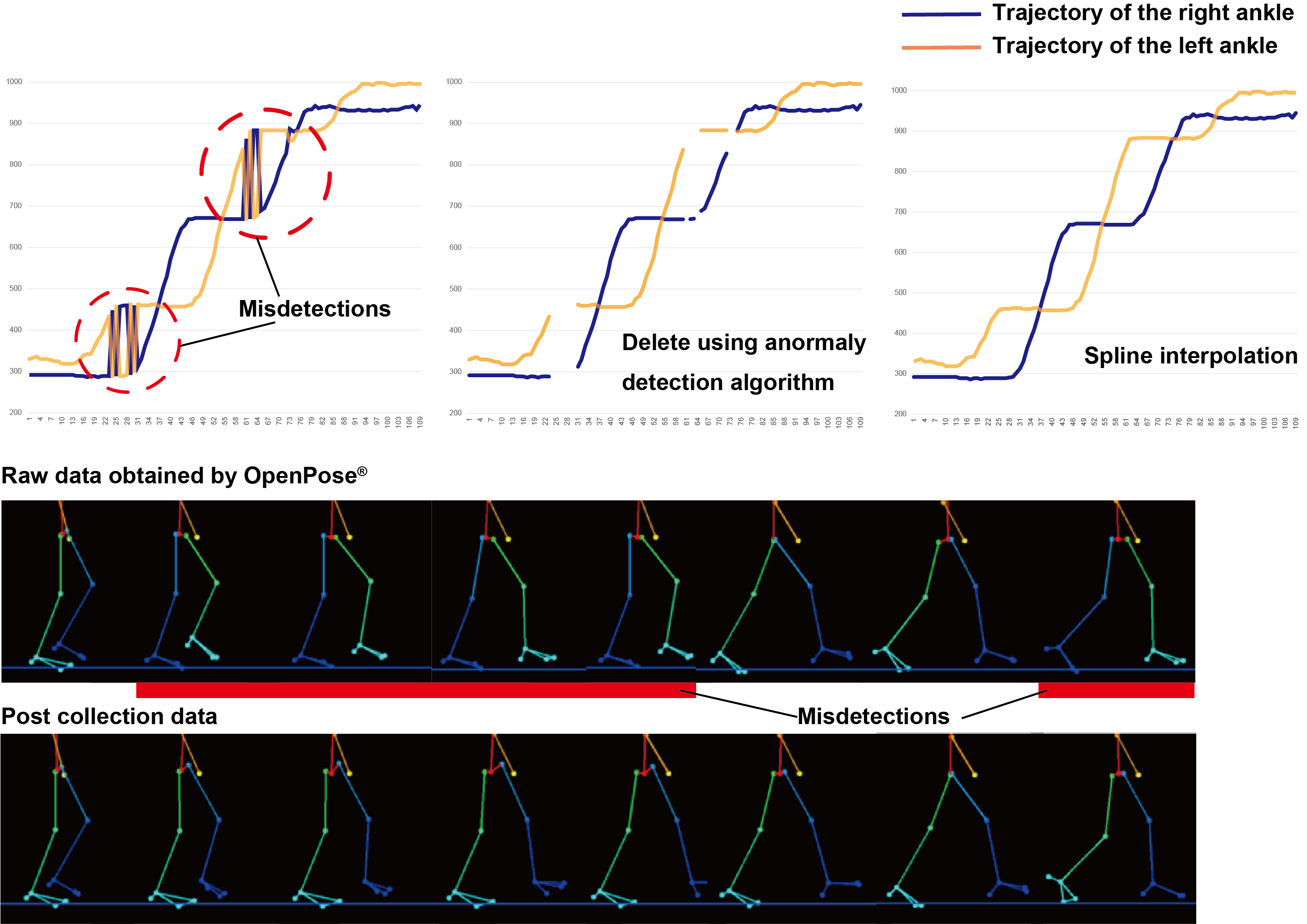
**

**Supplementary Figure S2: SHAP values of the selected features of the LightGBM model**

LightGBM, Light Gradient Boosting Machine; SD, standard deviation; SHAP, SHapley Additive exPlanations.

**
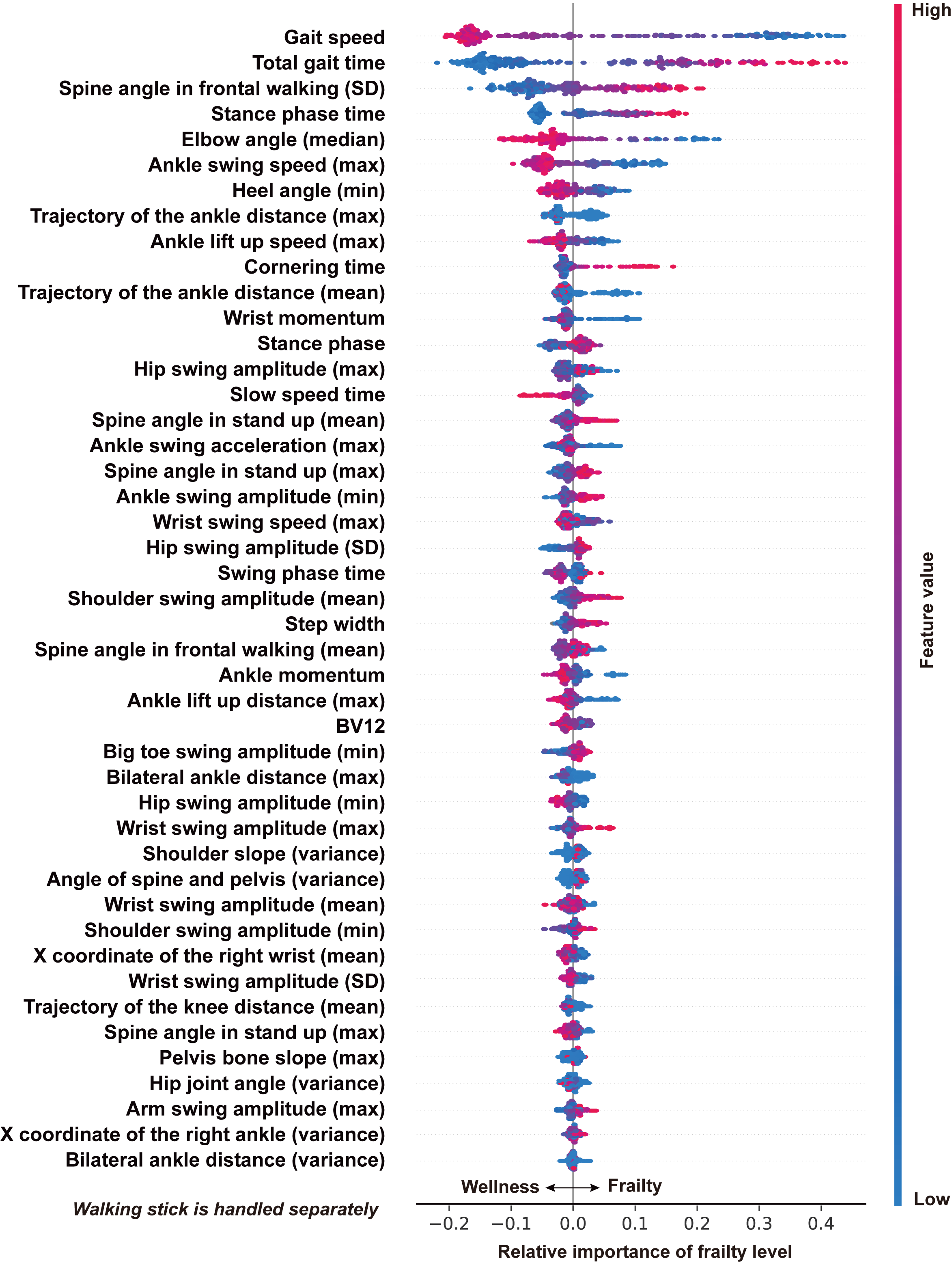
**
